# Development of Biocomposite Alginate-Cuttlebone-Gelatin 3D Printing Inks Designed for Scaffolds with Bone Regeneration Potential

**DOI:** 10.3390/md20110670

**Published:** 2022-10-26

**Authors:** Filis Curti, Andrada Serafim, Elena Olaret, Sorina Dinescu, Iuliana Samoila, Bogdan Stefan Vasile, Horia Iovu, Adriana Lungu, Izabela Cristina Stancu, Rodica Marinescu

**Affiliations:** 1 Advanced Polymer Materials Group, Faculty of Chemical Engineering and Biotechnologies, University Politehnica of Bucharest, 1-7 Gh. Polizu Street, 011061 Bucharest, Romania; 2 Zentiva S.A., 50 Theodor Pallady, 032266 Bucharest, Romania; 3 Department of Biochemistry and Molecular Biology, University of Bucharest, 91-95 Splaiul Independentei, 050095 Bucharest, Romania; 4 Research Institute of the University of Bucharest, 050663 Bucharest, Romania; 5 National Research Center for Micro and Nanomaterials, Faculty of Chemical Engineering and Biotechnologies, University Politehnica of Bucharest, 060042 Bucharest, Romania; 6 National Research Center for Food Safety, Faculty of Applied Chemistry and Materials Science, University Politehnica of Bucharest, 060042 Bucharest, Romania; 7 Academy of Romanian Scientists, 54 Splaiul Independentei, 050094 Bucharest, Romania; 8 Faculty of Medicine, Department of Orthopedics, University of Medicine and Pharmacy “Carol Davila” Bucharest, Eroii Sanitari Street No. 8, District 5, 050474 Bucharest, Romania

**Keywords:** alginate, cuttlebone, gelatin, thick paste-like ink, 3D printing, composite scaffolds, bone

## Abstract

Fabrication of three-dimensional (3D) scaffolds using natural biomaterials introduces valuable opportunities in bone tissue reconstruction and regeneration. The current study aimed at the development of paste-like 3D printing inks with an extracellular matrix-inspired formulation based on marine materials: sodium alginate (SA), cuttlebone (CB), and fish gelatin (FG). Macroporous scaffolds with microporous biocomposite filaments were obtained by 3D printing combined with post-printing crosslinking. CB fragments were used for their potential to stimulate biomineralization. Alginate enhanced CB embedding within the polymer matrix as confirmed by scanning electron microscopy (ESEM) and micro-computer tomography (micro-CT) and improved the deformation under controlled compression as revealed by micro-CT. SA addition resulted in a modulation of the bulk and surface mechanical behavior, and lead to more elongated cell morphology as imaged by confocal microscopy and ESEM after the adhesion of MC3T3-E1 preosteoblasts at 48 h. Formation of a new mineral phase was detected on the scaffold’s surface after cell cultures. All the results were correlated with the scaffolds’ compositions. Overall, the study reveals the potential of the marine materials-containing inks to deliver 3D scaffolds with potential for bone regeneration applications.

## 1. Introduction

Implantation of bone grafts is a frequent routine in clinical practice [1], however, synthetic grafting materials have displayed various limitations when used in hard tissue regeneration and repair. Hence, researchers have shifted their attention towards natural resources developing a diversity of approaches for new functional biomaterials. The fabrication of high-precision 3D structures by mimicking the microstructural and morphological characteristics of tissue-specific extracellular matrices (ECM) is one of the main research directions in the development of scaffolds for tissue engineering and regenerative medicine (TERM) [2]. The ECM of hard tissues represents a multi-component microenvironment whose specificity and complexity remain challenging to reproduce even with sophisticated approaches and synthetic materials. To compensate for this, natural materials that are often bioactive are widely investigated as building blocks with cooperative properties for the fabrication of multi-material bone-mimetic scaffolds. From a different point of view, the interest in low-cost and abundantly available natural resources with potential to be converted into biomedical products has increased considerably [3]. For instance, various species of marine algae are used as sustainable sources for biopolymer production, such as alginates that are extracted from brown algae [4]. In particular, sodium alginate (SA) is a biocompatible linear polysaccharide that has been largely explored for tissue repair and regeneration due to its low toxicity and mild ionic gelation when in contact with multivalent cations [5,6,7,8,9,10,11,12,13]. This polysaccharide also received extensive attention in formulating inks and bioinks due to its capacity to experience gelation under cell-friendly conditions [9,10,12,14,15]. Other marine species such as corals and cuttlefish represent abundant sources of calcium carbonate [1]. The cuttlefish skeleton, cuttlebone (CB), is appealing for bone regeneration due to its inexpensiveness, easily availability from all the seas of the world, osteoconductivity, high osteoinductive potential, and chemical and structural compliance to human bone tissue [1,16]. CB consists of an external dorsal shield and an internal lamellar matrix that is primarily composed of 87.3–91.75% calcium carbonate (aragonite phase) associated with 3–4.5% organic components (mainly β-chitin) [3,17,18,19]. Combining SA and CB with gelatin was preliminary investigated by our group to explore the potential of combined properties for bone regeneration scaffolds and 3D printing inks [20,21]. Gelatin is widely used as collagenous component of synthetic ECM-mimetic scaffolds [13,22,23]. Gelatin from cold water fish skin (FG) is promising in ink formulations [24,25,26] as reported in our previous studies [21,27,28] and it allows for the preparation of more concentrated aqueous solutions [29,30] when compared to mammalian gelatins.

Bioinspired bone grafts based on calcium carbonates and hydrogel matrices were widely explored due to their potential of replicating features of the native hard tissue ECM [31]. The development of such biocomposites improves the mechanical behavior when comparing their simple hydrogel counterparts, which are known to be often insufficient for hard tissue applications, also enhancing the osteoconductive properties and stimulating bone tissue development [3].

Furthermore, 3D printing has shown considerable advantages in TERM over the traditional fabrication methods by producing advanced 3D scaffolds that closely match the predesigned model. Shape fidelity and resolution of 3D printed structures are important parameters that go hand in hand with an ink’s printability [5,32]. 3D printing with hydrogels based on natural polymers is typically associated with drawbacks such as insufficient self-support during printing and inappropriate stability and mechanical strength post-printing [32,33]. Various strategies have been reported to address this challenges, such as combining different biomaterials for a better consistency of the hybrid ink [9,32,34], developing more efficient crosslinking methods [9,24,33,35,36,37,38], or modifying the 3D printing set up [14,38,39,40].

The interactions between SA and CB through the partial crosslinking due to sodium–calcium ion exchange [21], and the use of a concentrated FG solution [27] may address the printability challenges of alginate-containing inks in overcoming experimental complications, such as strand fusion or scaffold collapse. It also provides the fabrication of biomimetic composite 3D scaffolds with improved printing resolution and controlled architecture.

Therefore, thick paste-type inks based on SA and CB mixed with FG were formulated for the fabrication of 3D-printed scaffolds for bone tissue regeneration and repair. In a previous work, our team demonstrated the potential of a concentrated FG–SA–CB ink to 3D-print porous scaffolds interesting as bone substitute [21]. However, that work explored the printability and properties of a composition with a high SA content (23% wt%) and only 34% CB (wt%), while the cellular response remained unexplored. Moreover, the resulting scaffolds presented a degradation of approximately 37% after 28 days in phosphate-buffered saline (PBS) potentially correlated with alginate exposure due to its high concentration [21]. In this context, in the present work we intended to go one step further, combining the three building blocks in inks with an increased mineral content using approximately 72% CB (wt%), while SA was used in a lower concentration, below 1.5% (wt%). The development of three ternary compositions and of one control without SA offers the advantage of understanding the effect of SA on improving the printability, the stability and the cellular response of the 3D-printed scaffolds. Environmental scanning electron microscopy (ESEM) was used to assess the microstructure of the scaffolds and to inspect the in vitro mineralization and cell morphology. Micro-computed tomography (micro-CT) was performed to characterize the microstructural features and the architecture of the 3D printed biocomposite scaffolds as fabricated and after compression. Cell-responsiveness was in vitro tested.

## 2. Results and Discussion

**Figure 1:**
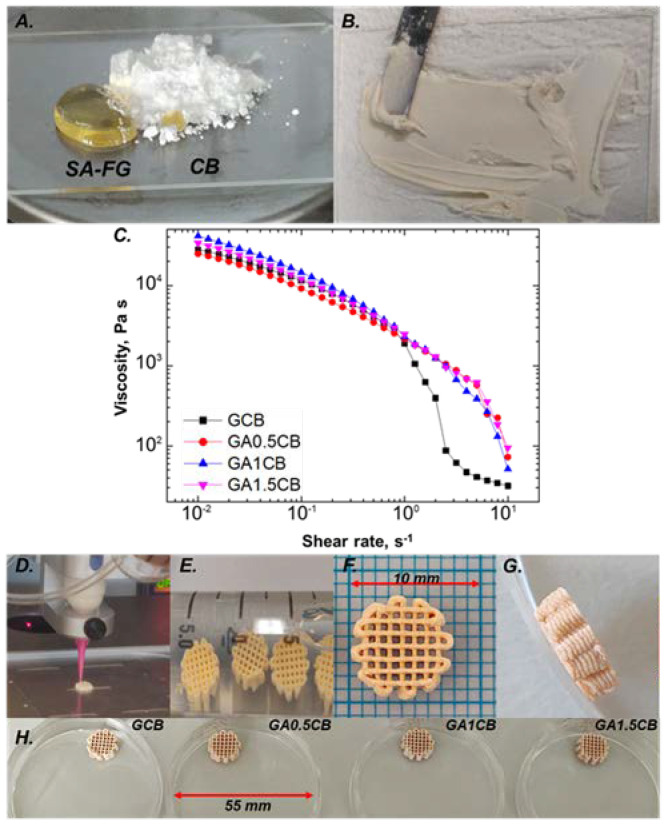
Representative digital images revealing the fabrication of 3D-printed scaffolds: (**A**) biopolymers solution and CB powder; (**B**) freshly prepared paste precursor; (**C**) shear viscosity investigation of the formulated inks: SA addition modifies the shear thinning behavior; (**D**) printing of one scaffold; (**E**–**G**) representative digital images of 3D-printed scaffolds obtained using a GA1CB precursor; (**H**) High resolution 3D scaffolds based on formulated inks.

The present study reports the formulation of thick-paste type inks designed for the fabrication of scaffolds with potential for hard tissue regeneration and repair. Considering the structural particularities of the aimed application, a bioinspired approach was proposed. To this end, starting from the compositional characteristics of bone ECM, calcium-carbonate-containing CB was selected as the mineral phase of the 3D printed composite scaffolds, gelatin as substitute for collagen, and alginate as equivalent for bone glycosaminoglycans. Figure 1A–H reveal representative digital images for the fabrication of the 3D-printed samples.

### 2.1. Rheological Tests

Insufficient resolution and shape fidelity remain key limitations for 3D-printable hydrogel precursors. Their improvement represents an important challenge, since the self-supporting characteristics of biopolymers during and after printing are still a concern to avoid the structure collapse or merging [41,42]. The development of inks with satisfying printability and self-supporting characteristics is highly dependent on the rheological properties of the precursor [42,43].

As depicted in Figure 1C, all compositions (see Table 1) present a shear thinning behavior, starting from a viscosity of over 10^4^ Pa s at low shear rates (10^−2^ s^−1^). Although the presence of SA does not significantly alter the pastes’ viscosity in the shear rate interval 10^−2^—10^0^ s^−1^, it modifies the rheological behavior when further increasing the shear rate. While fish gelatin/cuttlebone (GCB) ink presents a sudden viscosity drop at 1 s^−1^, followed by a slower decrease in the rest of the shear rate interval, fish gelatin/alginate/cuttlebone (GACB) precursors exhibit a sharp drop in viscosity at higher shear rates (2 s^−1^ for GA0.5CB and 4 s^−1^ for GA1CB, respectively). In the case of complex fluids, viscosity drops are assigned to the breakage of intermolecular forces. Therefore, this delay may be attributed to a lengthier rearrangement of the molecules in the three-component inks when compared to the GCB control composition, indicating that an increase of the alginate content leads to more stable inks. These findings were further confirmed during the 3D printing process, when higher SA content imposed a decrease in feed rate at the same pressure value, to obtain continuous filaments (see Table 2). This suggested that the increasing alginate content leads to additional intermolecular forces that restrict the mobility of the macromolecular chains and that require more time to reorganize.

### 2.2. Scaffold’s Manufacturing Process

As shape fidelity, along with scaffolds’ integrity and reproducibility, during 3D printing can dictate the overall performance of the scaffolds, 3D printing experiments were performed to identify the adequate processing conditions, also considering the precursors homogeneity. Hernández-Sosa et al. have clearly highlighted the importance of the adequate rheological properties and optimized printing parameters to further deliver easy-to-handle 3D scaffolds. Preventing droplet formation or nozzle clogging, avoiding collapse/fusion, and the formation of continuous and uniform filaments for stacking multiple layers, with the optimal height of the layers, are mandatory for achieving good shape fidelity [42].

The extrusion pressure and feed rate were adjusted to ensure the deposition of continuous filaments and to avoid the merging or collapsing caused by the deposition of thick filaments. The extrusion pressure was 550 ± 50 kPa, while the feed rate was adjusted between 1.8 and 3.5 mm/s, depending on the composite precursor used. As previously mentioned, lower feed rates were required for the SA-containing precursors to deposit continuous filaments. The established parameters for all four inks are indicated in Table 2. The choice of the G25 nozzle was considered the most useful for the printability of the prepared precursors. Layer thickness was set at approximatively 75% of the G25 nozzle diameter. This is also a crucial parameter for the quality of the 3D-printed scaffolds, which considerably influences the height of the printed scaffold and the z-resolution. In addition, the concordance between printing time corresponding to one layer and the drying time of the filaments was important, to allow for the support needed for the next layers and to ensure the stability and integrity of the scaffold. A certain mechanical strength of the ink is essential to self-support immediately upon extrusion and to retain the desired shape [44]. This is often resolved by combining different biomaterials for a better consistency of the ink, using crosslinking through immersion in a crosslinking bath or photo-crosslinking. The current study reports the development of thick paste-type inks to fabricate high resolution 3D scaffolds, without using crosslinking protocols during 3D printing to stimulate their shape retention ability. Previous works of our group reported the formulation of thick paste-type inks containing FG and SA [27], FG, SA and CB [21], and FG and wollastonite [28]. The formulations from [21] involved the development of 3D printing inks based on the incorporation of CB and a high alginate fraction (as powders) in the FG protein solution (FG:CB:SA mixed in a mass ratio of 43:34:23), to stimulate shape fidelity. In the present research, we investigate a new system prepared using low concentrated aqueous SA solutions (0.5 wt.%, 1 wt.%, 1.5 wt.%). Therefore, the fabrication protocol including components concentration, printing parameters, crosslinking treatment, are different than in the previous study [21]. Moreover, considering the high SA concentration in [21], it deserves to be mentioned that both matrix reorganization as well as uncrosslinked or weakly crosslinked SA have determined a partial degradation, and a decrease with 62.8% of Young modulus of degraded scaffold (after incubation 28 days in PBS). Therefore, it appears important to explore here a new ternary system with lower SA content.

### 2.3. Crosslinking Efficiency Investigation

**Figure 2:**
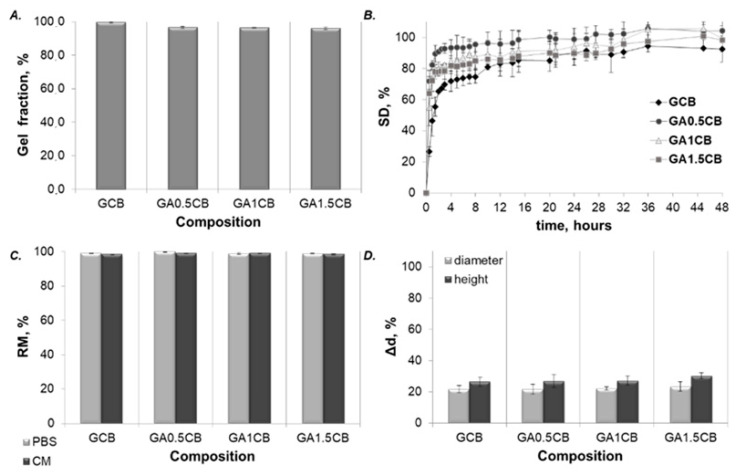
(**A**) GF results; (**B**) Swelling behavior of the 3D-printed scaffolds in phosphate-buffered saline (PBS); (**C**) Remaining mass (RM) of the scaffolds after incubation in culture medium (CM) and PBS; (**D**) Dimensional changes (Δd, %) of the 3D-printed scaffolds in diameter and height at the equilibrium point of swelling in PBS.

The composite samples were submitted to a post-processing crosslinking treatment to increase the stability and mechanical strength of the 3D-printed scaffolds by preventing the polymers’ dissolution in aqueous fluids. The external gelation of SA-based scaffolds in calcium baths is a common procedure of obtaining alginate hydrogels using cation diffusion, typically leading to a gradient of calcium content towards the surface [45]. In this work, both SA and FG were crosslinked by the diffusion of glutaraldehyde (GA) and calcium chloride from the scaffold surface to the inner layers in the presence of CB. The obtained filaments consisted of interpenetrating networks (IPNs) in calcium alginate and FG, containing immobilized CB fragments. In addition, increasing the amount of alginate in the matrix was expected to improve the dispersion of CB. The efficiency of the crosslinking method was explored by gel fraction (GF) (Figure 2A). The obtained values (above 96%) indicated a good stability of the scaffolds.

### 2.4. Structural and Dimensional Stability and Integrity

Scaffolds’ stability and integrity were estimated by determining their swelling behavior, the dimensional variations in their rehydrated stage and their degradation capacity. The immersion of the 3D-printed scaffolds in PBS caused changes in their weight and size, as indicated by their swelling degree (SD, %) and dimensional stability results presented in Figure 2B,D. Although the swelling capacity (Figure 2B) varied depending on the composition, only minor differences were observed. GCB scaffolds have reached the smallest maximum swelling degree (MSD, %) (94.3 ± 3.7%) at their equilibrium point (36 h) compared to the other alginate-containing compositions for which the MSD (%) was slightly higher, around 100%. At 36 h, GA0.5CB and GA1CB scaffolds reached an MSD (%) of 105.5 ± 2.3% and 105.0 ± 11.0%, respectively, while GA1.5CB scaffolds have reached 100.7 ± 7.5% at their equilibrium point of 45 h. Alginate addition slightly increased the water affinity when compared to GCB, due to the superabsorbent nature of this polysaccharide additive.

The stability and integrity of the 3D scaffolds were not altered during the 21 day incubation in CM or in PBS, with RM values above 98%, confirming the efficiency of the crosslinking method (Figure 2C). No differences between RM values were obtained. As mentioned above, in the previous study of our group, the 3D-printed scaffolds based on FG concentrated solution and the mixed SA–CB powder reached a mass loss of about 6% after 7 days and about 37% after 28 days of incubation in PBS [21]. This may suggest that the interactions between the ink components is decisive for achieving a structurally and functionally optimized scaffold; in the present study, dissolving FG in SA solution and combining it with CB powder stabilized the composite network, providing an advantage over the procedure and ink developed in [21].

Furthermore, the dimensional variations of the 3D scaffolds are represented in Figure 2D, with smaller variations in the scaffolds’ diameter than in the scaffolds’ height for all four compositions. No significant differences were evidenced between the samples. The smallest variations for both dimensions (21.7 ± 2.4% in diameter and 26.5 ± 2.7% in height) were registered for the GCB scaffolds. These results may also be correlated with the lower swelling behavior of GCB. The use of alginate in the tricomponent formulations slightly increased the size changes. This feature may be associated with the SA increasing the water affinity and facilitating CB’s redistribution. We have previously shown that water affinity is determined by the mineral type, and the internal porous structure of CB (remained after grinding) may also increase water affinity [20].

Overall, the results for swelling capacity, dimensional variations, and degradation experiments have highlighted an advantageous stability of the GCB and GACB scaffolds in aqueous fluids.

### 2.5. Morpho- and Microstructural Characterization

**Figure 3:**
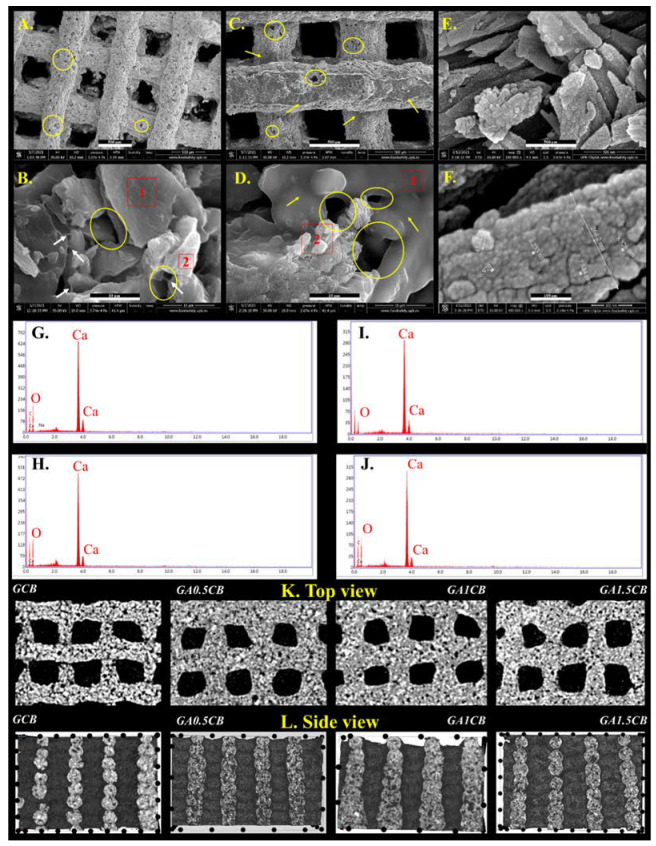
Representative ESEM micrographs revealing the morphology of GCB (**A**,**B**), GA1.5CB (**C**,**D**) scaffolds and CB particles (**E**,**F**) (yellow circles—pores; yellow arrows—hydrogel layer coating CB particles; white arrows—hydrogel bridges; scalebars: (**A**,**C**,**E**)—500 µm; (**B**,**D**)—10 µm; F—100 nm); EDAX spectra of: (**G**)—area 1 in micrograph (**B**); (**H**)—area 2 in micrograph (**B**); (**I**)—area 1 in micrograph (**D**); (**J**)—area 2 in micrograph (**D**); representative micro-CT details from orthogonal views: (**K**)—top view; (**L**)—side view.

Morpho- and microstructural characterizations were performed by ESEM and micro-CT, confirming the obtaining of macroporous scaffolds with continuous filaments forming square-shaped open pores. ESEM micrographs revealed that all scaffolds had a biphasic structure, with composite filaments consisting of numerous CB particles homogeneously embedded within an amorphous hydrogel phase; representative micrographs are shown for GCB (Figure 3A,B) and GA1.5CB (Figure 3C,D). An interesting element to consider regarding the scaffolds’ morphology is represented by the changes of the particles’ surface appearance after the addition of alginate. In GCB, the CB irregular microparticles were covered by a thin amorphous gelatin layer leading to a relatively smooth surface following the CB’s nanostructured topography. The nanostructured surface of pristine CB microparticles is shown in Figure 3E,F and served as a control. Alginate addition improved the continuity of the 3D-printed filaments, providing better embedment of CB fragments, and thicker hydrogel islands (Figure 3C,D). Figure 3D exposed the nanostructured surface of CB only in isolated areas of the scaffolds due to the thicker hydrogel coatings for CB particles in GA1.5CB filaments.

Small micropores may be noticed at the surface of the filaments, most probably due to CB, solvent evaporation, and matrix reorganization during drying prior to ESEM analysis (Figure 3A–D). Furthermore, EDAX spectra confirmed calcium-rich areas all over the scaffolds (Figure 3G–J). Unlike the ESEM analyses that require dry samples due to the applied vacuum, the micro-CT allows the analysis of hydrated scaffolds, thus granting the assessment of their architectural features in a more body-like environment. The registered micrographs permitted the observation of the microstructural details, revealing the composite nature of the fabricated scaffolds; in addition to the macroscale porosity as set by the 3D printing fabrication, the scaffolds also display numerous interparticle pores obtained through the arrangement of the CB particulates in the SA-FG matrix during extrusion. Details regarding the above-mentioned composite-specific and distinct porosity may be observed in Figure 3K,L (side view and top view images of the scaffolds) as well as in Supplementary Video S1 to S4 (supplementary material).

Serra et al. proposed 3D-printed scaffolds containing polylactic acid and glass particles, and studied the morphological and microstructural aspects also using SEM and micro-CT [46]. The composite scaffolds also displayed a combination of porosities considering the macroscale architecture (macropores) and micro and nanopores appeared as a result of solvent evaporation [46].

Both SEM and micro-CT have exposed relevant microstructural features in a previous study of our group, related to the 3D printing of macroporous scaffolds with open pores, the distribution of polysaccharide in the matrix (potential reinforcing additive), and the stability of architectural characteristics during dehydration/rehydration [27].

### 2.6. Mechanical Behavior

The data on the mechanical properties of hydrogel-type 3D-printed scaffolds based on gelatin and alginate vary considerably depending on the gelatin origin, polymer content, crosslinking procedure, and scaffold architecture. Cheng et al. fabricated 3D-printed structures based on a mixture of 1% (*w*/*v*) alginate and 3% (*w*/*v*) gelatin, and achieved a Young’s modulus of 20.4 kPa [47]. You et al. obtained 3D structures with different orientations and strand spacings using a mixture of 6% (*w*/*v*) gelatin and 3% (*w*/*v*) alginate; the structures with 0.8 mm strand spacing obtained a Young’s modulus of 92 kPa, while those with 1 mm strand spacing registered a Young’s modulus of 54 kPa [23].

**Figure 4:**
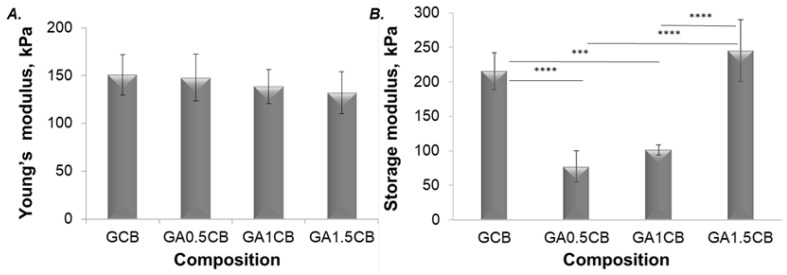
Results of the mechanical investigation: (**A**) Bulk compression testing—representative dependency of the Young’s modulus on the SA loading of the 3D-printed scaffolds; (**B**) Nanoindentation—representative results of the nanoindentation test revealing the effect of SA on the indentation storage modulus (G’), statistical significance: *** *p* < 0.001, **** *p* < 0.0001.

The mechanical characteristics of a hydrogel scaffold are often a main challenge in the fabrication of scaffolds for tissue reconstruction or regeneration. While the mechanical bulk properties are rather important for the application-related functionality, the micro-mechanical properties of the surface may influence the cellular adhesion or migration. In the present work, the effect of small amounts of SA on the Young’s modulus of the scaffolds was investigated, and the obtained results are presented in Figure 4A. The compression tests were performed without visually breaking the structure and all samples maintained their shape integrity. Using a low amount of SA in the ink did not modify the bulk elasticity of GA0.5CB with respect to GCB. Further increasing the SA content reduced the bulk Young’s modulus values of the hydrated scaffolds (Figure 4A). These results may be correlated with the highest structural variations reached by GA1.5CB scaffolds, indicating a more elastic hydrated scaffold due to alginate, in agreement with the higher hydration degree and dimensional variation.

Nanoindentation provided supplementary data to the bulk compression test, describing the localized surface response of the hydrated composite filaments at compression. As shown in Figure 4B, there is a visible discrepancy between the values for storage for each composition. The inks with the lowest and medium SA content led to a G’ decrease, corresponding to an increase in the samples’ surface elasticity when compared to GCB. However, G’ increased for the scaffolds with highest SA content. Possible matrix reorganization during compression, SA content, the presence of pores, CB displacement during incubation in a crosslinking bath, and calcium gradients expected to occur due to the diffusion-based crosslinking might account for these results.

**Figure 5:**
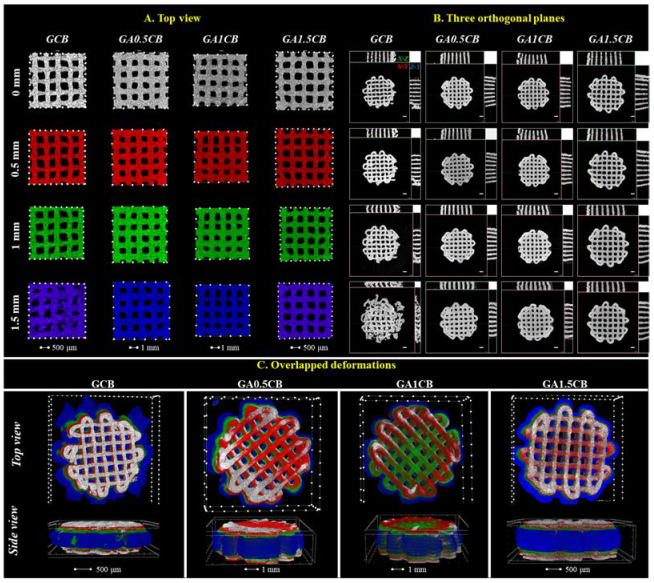
Representative micro-CT images of the GCB and GACB scaffolds at various compression points: 0 mm, 0.5mm, 1 mm, 1.5 mm: (**A**)—top view; (**B**)—in the three orthogonal planes (images registered in DataViewer); (**C**)—overlapped deformations as top view and side view (grey—0 mm; red—0.5 mm; green—1 mm; blue–1.5 mm).

Furthermore, micro-CT investigations revealed important data on the morpho- and microstructural changes of the hydrated scaffolds under controlled compression steps (Figure 5). First, the addition of SA has ensured better integrity and stability for the scaffolds during compression, noticeable especially when a 1.5 mm displacement (over 40% of scaffolds height) was applied. As depicted in Figure 5, while in the case of the SA-richest scaffold (GA1.5CB) a displacement of 1.5 mm has almost no impact on the microarchitectural features of the sample, the GCB control is completely crushed. The images obtained from both CTVox (Figure 5A) and DataViewer (Figure 5B) clearly indicate the different behavior under load between the control sample (GCB) and the alginate-containing samples (GACB). This aspect can be best noticed in the lateral view (Figure 5B), where the presence of alginate (all tricomponent samples) and its increasing content led to more dimensionally stable filaments that do not bend when load is applied. These data have confirmed the results obtained using the conventional macroscopic compression method (bulk Young’s modulus of the composite scaffolds). The increase in elasticity at compression with increasing alginate can be also noticed in the Supplementary Video S5–S8 (supplementary information). This behaviour is remarkable considering that SA represents the minority component in all GACB samples.

### 2.7. In Vitro Biocompatibility Investigation: Cell-Scaffolds Interactions

#### 2.7.1. Biocompatibility Investigation

**Figure 6:**
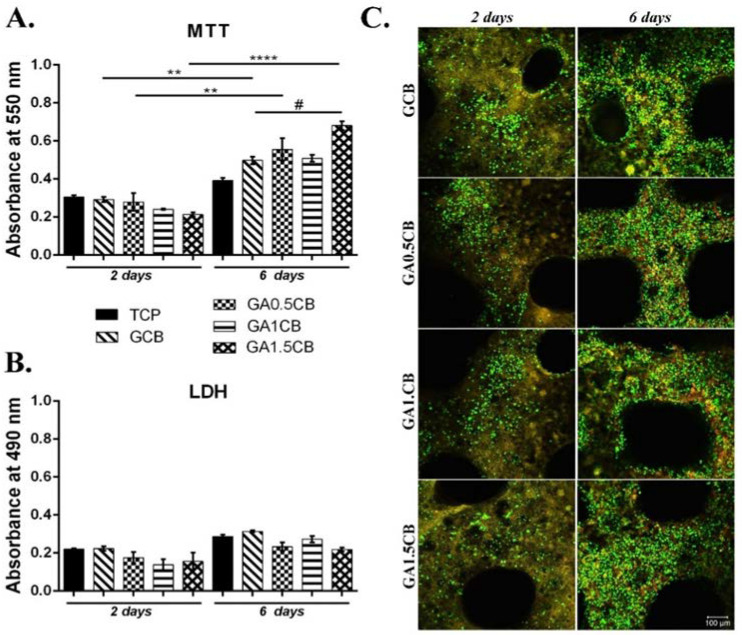
Biocompatibility evaluation of printed composites in contact with murine preosteoblasts: (**A**). Cell viability and proliferation by MTT assay at 2 and 6 days; Statistical significance: # *p* < 0.05, ** *p* < 0.01, **** *p* < 0.0001.; (**B**). Cytotoxicity by LDH test at 2 and 6 days; (**C**). Live/dead staining of cells and confocal microscopy images: live (green labeled with calcein AM) and dead (red labeled with ethidium bromide) cells; scale bar = 100 µm.

After 2 days of culture in classic conditions, the MTT profile revealed a similar cell viability for all the composites when compared to the tissue culture plastic control (TCP), with no significant differences between the four tested materials. Although no significant changes were observed, a slightly higher viability was detected on GA0.5CB when compared to GA1CB and GA1.5CB (Figure 6A). At 6 days of culture, the cells started showing different levels of proliferation, depending on the scaffold’s composition. The control system GCB showed a statistically significant higher proliferation (*p* < 0.001) at 6 days of culture when compared to the level registered at 2 days. From 2 to 6 days of culture, cell proliferation was also significantly higher on GA0.5CB (*p* < 0.01) and GA1.5CB (*p* < 0.0001). Moreover, even though no statistical significance was noticed after 6 days of culture on GA1CB when compared to GA0.5CB and GA1.5CB, a slight tendency of increased proliferation compared to GCB was detected. When comparing the proliferation at 6 days on all tested scaffolds, cells on GA1.5CB showed the most statistically significant increase (*p* < 0.05) when compared to GCB, suggesting a beneficial effect of increasing the alginate concentration in the inks. Although alginate is not a cell-adhesive biomaterial [48,49], its presence in the IPNs seems to improve CB distribution, biomaterial organization and interaction with cells. Neves et al. have focused on the useful and attractive characteristics of alginate in developing multiple and complex alginate-based systems in the biomedical field, and highlighted that alginate remains one of the most frequently used natural-based polymers [48]. The possibility of modulating different types of interactions in the presence of CB might account for such behavior, and further studies will deeper explore these aspects.

The LDH assay indicated a reduced toxicity after 2 days of culture on all tested samples, similar to the TCP and GCB controls (Figure 6B). At 6 days, LDH enzyme release slightly increased for all tested systems, but remained at a similar level as GCB. However, low levels of cytotoxicity remained constant throughout the 6 days of culture, with no significant differences between 2 and 6 days on either scaffold. These results indicated that the tested composites do not cause the significant cell death of murine preosteoblasts during 6 days of culture in normal conditions.

Confocal microscopy images obtained after live/dead staining strengthened the MTT and LDH assay results, as a high fraction of live cells was observed after being in contact with all the composites (Figure 6C). Significant proliferation rates were revealed from 2 to 6 days of culture, as well as an evenly distributed cell model on the materials. The design pattern of these 3D scaffolds (0–90° deposition direction) has also allowed more surface exposure for the adhesion of the preosteoblast cells, as reported in the literature [50]. Additionally, visualization by confocal microscopy indicated an overall elongated cell morphology, characteristic for cell adhesion in the 3D culture systems. Thus, as live/dead, and MTT and LDH assays all indicated a good viability for cells cultured in contact with GACB materials, as well as low number of dead cells, these composites could represent a potential suitable scaffold for bone implantation.

#### 2.7.2. Cell Adhesion Investigation

**Figure 7:**
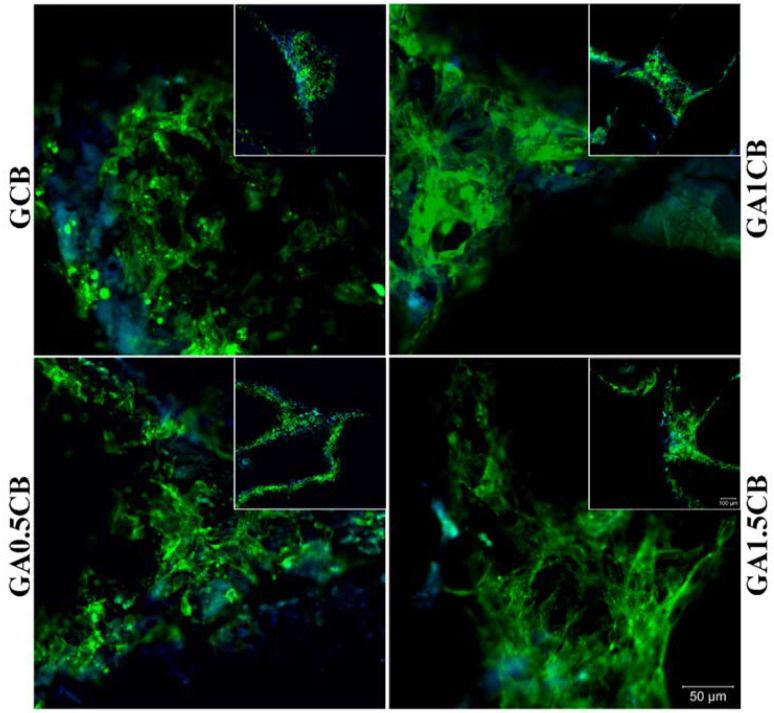
Cell adhesion assay performed after 48 h of MC3T3-E1 cell culture in contact with scaffolds. F-actin filaments (represented in green) developed by cells were stained with phalloidin–FITC, while nuclei (represented in blue) were stained with Hoechst 33341.

Phalloidin–FITC staining has revealed cells’ cytoskeleton by highlighting F-actin filaments, hence displaying a favorable overall cell adhesion on all the investigated scaffolds. However, there are some differences between the four tested composites and their ability to encourage cytoskeleton development. Cells that kept in contact with GCB scaffold have a more rounded phenotype, whereas F-actin filaments are clearly more elongated on the other three composites containing alginate (Figure 7). Even so, when comparing the actin filaments found on GA0.5CB, GA1CB and GA1.5CB, the longest and most visible filaments were observed on GA1.5CB, indicating that after 48 h, MC3T3-E1 cells had a better interaction with this scaffold. Thus, these results confirmed that a higher amount of SA in the scaffold’s composition was beneficial for cell adhesion, although it is acknowledged that alginate does not stimulate cell adhesion, nor protein adhesion [48,51]. Accordingly, it resulted that the cellular response was due to other factors such as the exposure of CB fragments in the alginate–gelatin IPNs also influencing the mechanical properties of the surface, as previously discussed. Neves et al. have pointed out an interesting aspect about the intrinsic bioinertness of alginate, which might be an advantage in different conditions, and the presence and density of cell adhesive moieties might be modulated by developing complex and combinatorial approaches [48]. Wattanaanek et al. have reported the fabrication of 3D-printed scaffolds based on alginate-containing multicomponent systems, obtaining suitable 3D morphologies of the scaffolds and mimicking ECM to stimulate osteoblast cell attachment and proliferation [50].

**Figure 8:**
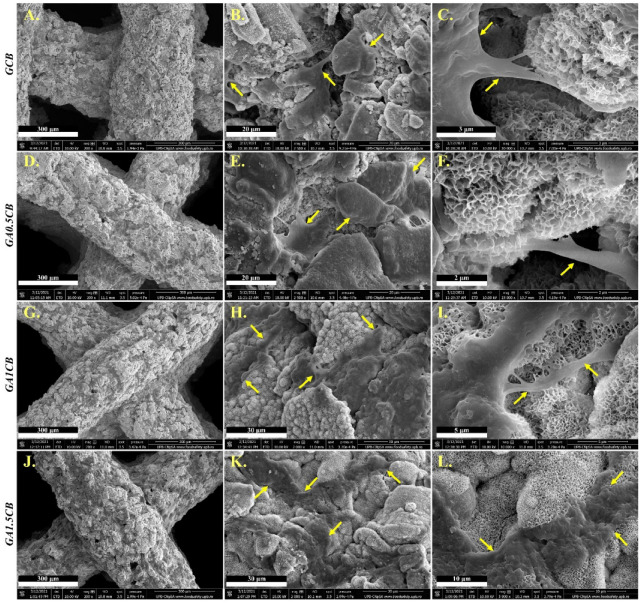
Representative ESEM micrographs of 3D printed scaffolds revealing cell attachment through focal adhesion: (**A**–**C**) for GCB scaffold, (**D**–**F**) for GA0.5CB, (**G**–**I**) for GA1CB and (**J**–**L**) for GA1.5CB scaffolds (yellow arrows indicating cells adhesion and spreading).

The above results were also confirmed by ESEM micrographs shown in Figure 8. These micrographs illustrate the cells’ adhesion and spreading on the surface of the 3D-printed scaffolds, revealing favorable cell–scaffold interactions as suggested by well-developed cellular elongations in connecting neighboring cells and anchoring on the nanostructured material. The cells’ spreading was enhanced on the alginate-richer scaffolds and the cellular bodies were thinner on GA1CB and GA1.5CB, spreading across a larger area and presenting more adhesion points (Figure 8H,K). Micrographs from Figure 8C,F,I,L have provided morpho-structural details indicating cell attachment through multiple focal adhesion to the printed scaffolds.

#### 2.7.3. Mineralization Analysis

In addition, the ESEM micrographs have evidenced an interesting change in the surface morphology of the scaffolds during cell culture conditions, indicating a strong mineralization. All the composite samples were covered by a new nanostructured mineral layer, with strongly adhered cells (Figure 8B,E,H,K). However, the new mineral was much denser on alginate-containing samples, while on GCB, the tablets and needles were freely grown from the composite particles’ surface (Figure 8). Increasing the amount of polysaccharide additive in GA1CB and GA1.5CB favored the development of denser semispheroidal deposits, and the mineral layer was thicker, more homogeneous and with CB fragments better embedded (Figure 8H,K). In comparison, the CB fragments with sharp edges observed on the GCB scaffold were covered only by a thin mineral layer and not so much embedded (Figure 8B). Bertuola et al. have also observed the formation of a carbonated hydroxyapatite layer on the scaffolds’ surface after being cultured for 2 days [13]. The addition of 45S5 bioglass gelatin/alginate/hyaluronic acid inks has favored the depositions of such inorganic materials [13].

To better understand the formation of such a mineral layer, an additional test was performed by incubating the scaffolds in acellular CM. A comparison between those incubated in acellular CM and in MC3T3-E1 murine preosteoblast-containing CM, respectively, was performed.

**Figure 9:**
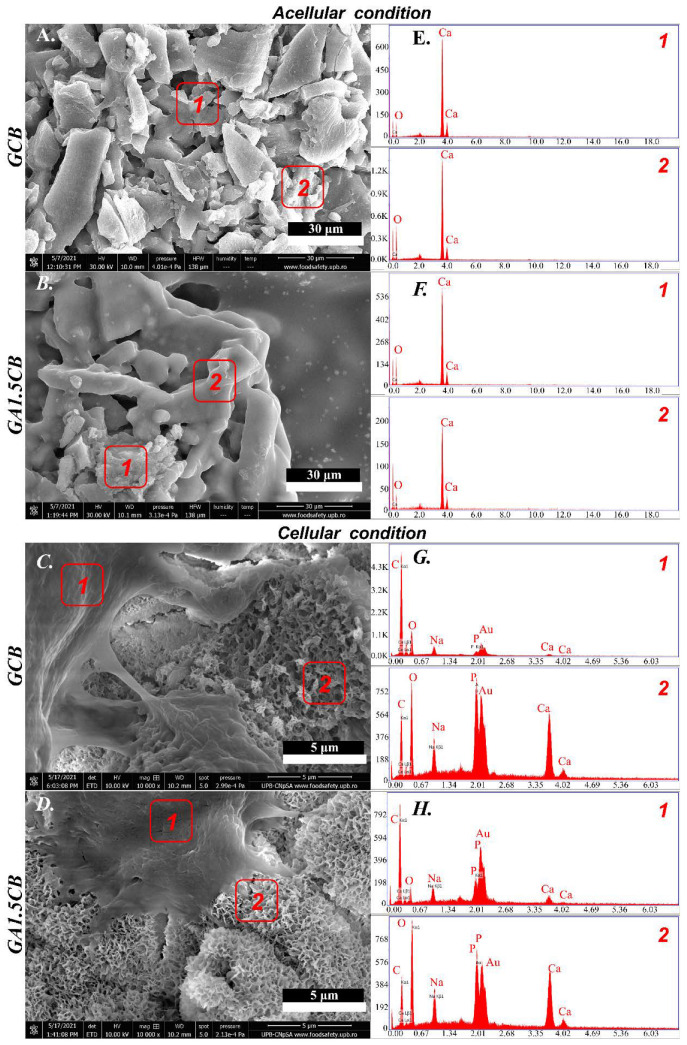
ESEM micrographs and EDAX spectra revealing morpho-structural details and element identification in selected areas of the scaffolds incubated in acellular ((**A**,**E**) for GCB and (**B**,**F**) for GA1.5CB) and cellular conditions ((**C**,**G**) for GCB and (**D**,**H**) for GA1.5CB), respectively (acellular condition: EDAX spectra corresponding to area 1 and 2—scaffold surface; cellular condition: EDAX spectra corresponding to area 1—cell surface, area 2—mineralized surface).

The micrographs in Figure 9 have illustrated that only the immersion in CM-containing preosteoblast cells facilitates the formation of such a new phase on the scaffold’s surface. The scaffolds incubated in acellular CM up to 7 days did not modify their topography (Figure 9A,B), nor their elemental composition—rich in calcium due to CB and crosslinked alginate (Figure 9E,F). The microstructural aspects shown in Figure 9A for the GCB scaffold were corelated with an amorphous structure characteristic to gelatin which coated the individual CB particles and only partly connected the CB fragments along the 3D-printed filament. The morphological aspects revealed by Figure 9C,D (cellular condition) and the phosphorus presence confirmed by representative EDAX spectra in Figure 9G,H suggested the formation of a new calcium phosphate phase (CaP) whose morphology might indicate an in vitro-synthesized apatite. The newly formed CaP was distinguished from the calcium carbonate from CB. An intimate contact was noticed between the cellular extensions and the newly formed nanostructured CaP phase on the GA1.5CB scaffold (Figure 9D). Two EDAX spectra (Figure 9G,H) were recorded for each sample, one on the cellular surface (area 1) and one on the new mineral phase (area 2). For GCB (Figure 9G), the spectra corresponding to the cell surface (area 1) presented a high amount of C element due to the cellular body and a Ca/P ratio of about 1.18, while the one from the mineralized surface (area 2) displayed a Ca/P around 1.60. For GA1.5CB (Figure 9H), the spectra corresponding to the cell surface (area 1) also presented a high amount of C element but a lower Ca/P ratio of around 0.97, while the spectra from the mineralized surface (area 2) displayed a Ca/P of about 1.91.

The above results indicate the beneficial interaction between these preosteoblasts and scaffolds, as well as the SA’s impact in forming new apatite phase (nanostructured CaP thinner tablets), predicting the potential of the GCB and GACB scaffolds for bone tissue regeneration. Such behavior may associate the study with the concept of 4D printing. A reconfiguration of the scaffolds’ surface in terms of composition and morphology was established in the presence of stimuli (preosteoblasts and SA), converting the scaffolds into different composites than those originally made. In this context, further studies are needed to validate this theory.

## 3. Materials and Methods

### 3.1. Materials

FG (cold-water fish skin gelatin), SA (alginic acid sodium salt from brown algae), 50% GA aqueous solution, calcium chloride and 0.01 M PBS (pH 7.4, prepared as indicated by the manufacturer) were supplied by Sigma-Aldrich. Ethyl alcohol (EtOH, 99.3%) was purchased from Chemical Company, and CB was purchased from pet stores (Romania). Distilled water (dH_2_O) was obtained using a GFL distiller apparatus.

### 3.2. Methods

#### 3.2.1. Preparation of CB Powder

The dorsal shield of the CB was detached, then the lamellar part was sectioned into smaller blocks which were extensively washed with dH_2_O, and then dried. Further, the CB blocks were grinded, and the resulted powder was washed three times with dH_2_O, separated through centrifugation, and subsequently dried and re-grinded using a mortar. The finest fraction (<300 µm) was collected by sieving. No treatments were applied for the organic content removal from CB.

#### 3.2.2. Ink Formulation

Aqueous solutions with increasing SA content (0.5 wt.%, 1 wt.%, 1.5 wt.%) were prepared, in which FG was incorporated under stirring at 40°C to a final 50 wt.% FG content. An FG 50 wt.% aqueous solution was also prepared as a control, without SA. The necessary amount of CB powder was incorporated stepwise in the weighed FG–SA solutions and control FG, respectively, at room temperature. Table 1 illustrates the four composite inks. For example, to prepare GA1CB, FG was incorporated in a 1 wt.% SA solution (up to 50 wt.% FG), and finally 10 g FG-SA solution were mixed with 13 g CB.

Using a method previously reported by our group [21,27,28], similarly to the preparation of orthopedic cements, the solid phase was carefully mixed with the liquid phase using a spatula and the formulation started to change its consistency and became finally a thick paste-type precursor. The mixing procedure was critical for the ink homogeneity, and the mixing time of approximatively 1–2 min being optimal to avoid the early drying of the polymeric formulation.

#### 3.2.3. Rheological Test

The rheology tests were performed using a Kinexus Pro rheometer equipped with a plate–plate geometry and a Peltier element for precise temperature control. To suit the experimental conditions during 3D printing, the measurements were performed at 25 °C, immediately after ink preparation; a water lock was used to avoid the precursor dehydration during the tests. The paste-type inks were fixed on the lower plate of the rheometer, and the upper plate was lowered to a fix gap of 0.5 mm. The flow curve was registered at steady state in the shear rate interval of 10^−2^–10^1^ s^−1^. The obtained data were plotted in logarithmic scale.

#### 3.2.4. 3D Printing

GCB and GACB scaffolds were produced using the direct-dispensing printhead (Direct Dispenser DD135N) of a 3D Discovery Bioprinter (RegenHU, Villaz-St-Pierre, Switzerland). The inks were loaded in 3 mL syringes, and conical nozzles of 0.25 mm inner diameter (G25) were used. The 3D scaffold design was generated in BioCAD software (developed by RegenHU, Switzerland) as a cylindrical model with 8 mm diameter, 1 mm distance between two neighboring filaments, 8 layers of 0–90° deposition direction, and a layer thickness which represented 75% of the nozzle diameter used. The adequate processing parameters were determined through multiple 3D printing experiments and are listed in Table 2. The 3D scaffolds were printed on a plastic sheet at room temperature and kept in the refrigerator before crosslinking.

#### 3.2.5. Post-Printing Crosslinking Method

A crosslinking solution containing 1.5% (*w*/*v*) calcium chloride and 1% (*v*/*v*) GA was prepared in H_2_O:EtOH (1:1 volume ratio). The 3D-printed structures were maintained up to 72 h in the double crosslinking bath, for the stabilization of both polysaccharide and protein. Crosslinking took place through the diffusion of GA and calcium chloride from the scaffold surface to the inner layers. After crosslinking, the 3D structures (except those for gel fraction) were extensively washed with distilled water up to 24 h to eliminate the unreacted crosslinking agents, and then dried to a constant mass.

### 3.3. Characterization Methods

#### 3.3.1. GF Analysis

The 3D scaffolds were incubated up to 48 h in dH_2_O at 37 °C for the extraction of soluble fraction and to evaluate the crosslinking efficiency. The test was performed in triplicate and GF (%) was computed using Equation (1):

(1) $$\mathrm{GF},\;\%=\frac{w_{\mathrm{fi}}}{w_{\mathrm{in}}}\times 100$$

where w_in_ is the weight of dried crosslinked samples before extraction in dH_2_O and w_fi_ is the weight of dried samples after extraction in dH_2_O.

#### 3.3.2. Swelling Behavior Evaluation

The swelling capacity of the 3D scaffolds was investigated by incubation in PBS up to 48 h, at 37 °C, and was performed in triplicate. SD (%) was established gravimetrically using Equation (2):

(2) $$\mathrm{SD},\;\%\;=\frac{w_{t}-w_{i}}{w_{i}}\times 100$$

where w_i_ evidence the initial weight of dried specimen prior to PBS immersion and w_tt_ evidence the weight of the swollen specimen at pre-established immersion time, t. MSD (%) was considered at the equilibrium point.

#### 3.3.3. Structural Stability Test in PBS

The dimensional stability of the 3D scaffolds in aqueous fluids was investigated (in triplicate), by measuring the scaffolds dimensions (diameter and height) in both states, dried and rehydrated in PBS at swelling equilibrium point, respectively, using a digital caliper. A graphical representation was plotted based on the Δd (%) of diameter and height which were estimated using the Equation (3):

(3) $$\Delta d,\;\%\;=\frac{d_{s}-d_{d}}{d_{d}}\;\times 100$$

where d_d_ is the diameter/height of the dried specimen and d_s_ is the diameter/height of the swollen scaffold.

#### 3.3.4. Stability Test in CM and PBS

The stability of the 3D scaffolds was investigated (in triplicate) by keeping the samples in individual test tubes containing 10 mL of CM (10% fetal bovine serum and 1% antibiotic) or PBS (0.01 M, pH 7.4), respectively, up to 21 days at 37 °C. RM (%) was calculated using Equation (4):

(4) $$\mathrm{RM},\;\%=\frac{w_{\mathrm{df}}}{w_{\mathrm{di}}}\times 100$$

where w_di_ is the weight of dried specimen before incubation in CM or PBS and w_df_ is the weight of dried specimen after incubation.

#### 3.3.5. Morpho-Structural Characterization of 3D Printed Scaffolds

Morpho-structural characterization of the 3D-printed scaffolds was performed through ESEM, and micro-CT. ***ESEM micrographs*** were recorded on gold-sputtered dried samples using a focused ion beam—scanning electron microscope (FIB-SEM) system (model Versa 3D, Thermo Fisher Scientific, Co., Waltham, MA, USA). The ETD and CBS detectors have facilitated the simultaneous recording of signals acquired by secondary electrons (SE) and backscattered electrons (BSE). The surface of the samples (0° tilt) was examined in High-Vacuum (6.1 × 10^−4^ Pa) using a working distance of 9 mm and an accelerating voltage of 10 kV, for exposing topographical and compositional data.

***Micro-CT imaging*** was performed using a SkyScan 1272 high-resolution X-ray microtomograph (Bruker MicroCT, Kontich, Belgium) equipped with a mechanical testing stage of 440N. The projections were recorded at a camera binning of 2 × 2 with a voltage of 70 kV and an emission current of 140 µA, using a 0.5 mm Al filter. All samples were scanned at a pixel size of 8.5 μm with 3 average frames at every 0.3° angle step and an exposure time of 1000 ms.

#### 3.3.6. Mechanical Properties Evaluation

The mechanical properties of the equilibrium-rehydrated 3D-printed scaffolds were investigated using a conventional macroscopic compression method and at microscale, through indentation tests. In addition, micro-structural changes appeared under compression load were investigated using micro-CT.

***Compression*** behavior was tested using a Brookfield CT3 texture analyzer with a 4500 g cell load (Brookfield Engineering). The tests were performed in triplicate for each composition and the dimensions of the swollen scaffolds were measured using a digital caliper. The compression speed was set at 0.1 mm/s, and the swollen samples were uniaxial pressed by the mechanical cell. A stress deformation curve was plotted, and Young’s modulus was determined based on the slope of the initial linear part of compression curve, at a strain of 1%.

***Mechanical properties at surface*** have been locally investigated through indentation tests using a G200 Nano Indenter system (KLA Instruments, Milpitas, CA, USA) equipped with CSM and DCM II options. Seven indentations were performed along filaments with a diamond flat ended cylindrical tip (punch diameter of 100 μm) assuring at least 400 μm distance between them. The “G-Series DCM CSM Flat Punch Complex Modulus, Gel” method implemented in NanoSuite software was used to determine storge modulus G’ at a 10 Hz testing frequency, 5 μm indentation depth and 500 nm oscillation amplitude. Results were calculated for 0.5 Poisson’s ratio and are expressed as mean ± standard deviation.

***Deformation under compression*** was explored using the micro-CT method described in the Section 3.3.5. In addition to an initial scan performed in the absence of any load, the samples were also scanned at three pre-established compression points, i.e., 0.5, 1, and 1.5 mm, respectively. The compression speed was set at 1.5 µm/s and 15 min were allowed for the samples to stabilize under load previous to scanning. The projections were reconstructed using the NRecon software (version 1.7.1.6) and the obtained cross-sections were further utilized to obtain 3D images of the scanned samples using CTVox software (version 3.3. or 1403). To monitor the deformation of the filaments, 2D images of scaffolds were visualized in all three orthogonal planes, using the DataViewer (version 1.5.4.6) software.

#### 3.3.7. In Vitro Assessment of Biocompatibility

The composite scaffolds were tested for their biological effect on MC3T3-E1 murine preosteoblast cell line (ATCC CRL-2593, ATCC, Manassas, VA, USA), investigating the influence of both biogenous mineral component as well as the effect of SA amount. The samples were sterilized for 24 h by being exposed to UV light. After this, the samples were placed in 48-well culture plates with complete CM.

##### Achievement of Three-Dimensional Cell-Scaffold System

MC3T3-E1 murine preosteoblast were seeded on GCB, GA0.5CB, GA1CB and GA1.5CB composites in 48-well culture plates at a density of 2 x 10^5^ cells/cm^2^. Cells were allowed 24 h for adhesion and the forming of cell–scaffold systems. The constructs were maintained in standard culture conditions (37 °C, 5% CO_2_ and humidity) for 6 days, during which biocompatibility assays were carried out at 2 and 6 days.

##### Evaluation of In Vitro Biocompatibility

***MTT assay*** has evaluated the metabolic activity of living cells in contact with the scaffolds. A solution of methylthiazolyldiphenyl tetrazolium bromide (MTT, Sigma-Aldrich Co, Steinheim, Germany) was prepared at a concentration of 1 mg/mL in CM, according to manufacturer’s instructions. After 4 h of incubation with MTT solution, the resulted formazan crystals were solubilized using isopropanol, resulting in a violet solution. The final solution was measured at 550 nm, using a FlexStation3 spectrophotometer (Molecular Devices, Foster City, CA, USA).

***LDH assay*** has quantified the cytotoxic effect of the scaffolds exerted on the cells. The test was performed using TOX7 kit (Sigma-Aldrich Co, Steinheim, Germany), according to the protocol indicated by the manufacturer, and the final solution was measured at 490 nm, using the FlexStation3 spectrophotometer.

***Live***/***Dead assay*** is a qualitative test which helps the simultaneous staining of living and dead cells using Live/Dead kit (Invitrogen, Life Technologies, Foster City, CA, USA). The kit includes two components: calcein AM, which stains live cells in green, and ethidium bromide, staining dead cells in red. The examination was performed with a laser-scanning confocal microscope (Nikon A1/A1R Confocal Laser Microscope System) and the images obtained were analyzed using their corresponding software.

##### Cell Adhesion Investigation

To evaluate cell adhesion, F-actin filaments were stained 48 h after seeding. Cells were fixed for 1 h with a 4% paraformaldehyde solution (Sigma Aldrich Co, Steinheim, Germany). After fixation, the cell membrane was permeabilized for 1 h with 0.01% Triton X-100 solution (Sigma Aldrich Co, Steinheim, Germany) in bovine serum albumin (BSA). To diminish scaffolds’ affinity to fluorescence, cell–scaffold systems were incubated for 40 min with a 0.04% Trypan Blue solution, which was then thoroughly washed with PBS. Composites were incubated overnight with phalloidin–FITC (Sigma Aldrich Co, Steinheim, Germany) and for 20 min with Hoechst 33342 for cell nuclei staining (ThermoFisher Scientific, Foster City, CA, USA). Laser-scanning confocal microscope was also used for visualization and the images inspection was performed using microscope’s software. Additional details of cell morphology and spreading were acquired using the ESEM equipment described in Section 3.3.5.

#### 3.3.8. Statistical Analysis

The experiments were performed at least in triplicate (n = 3) and values obtained from the experiments are expressed as mean ± standard deviations. Statistical analysis was performed using the one-way ANOVA method followed by Bonferroni’s correction using GraphPad Prism Software 6.0. Statistically significant values were considered for *p* < 0.05.

## 4. Conclusions

3D printing thick biocomposite inks have been formulated to obtain porous bone scaffolds with cooperative structural and bioactive properties for bone restauration. The most important components of bone–ECM were mimicked using marine-origin resources: FG protein, SA polysaccharide and CB as a mineral phase. The 3D printing conditions were established, and a crosslinking treatment was applied post-printing. The results for gel fraction, swelling behavior, structural stability and degradation capacity have confirmed the role of alginate to obtain an advantageous stability of these composite scaffolds in aqueous fluids. In vitro biocompatibility assays showed that all composites had the ability to promote MC3T3-E1 murine preosteoblast adhesion, growth, and proliferation. This work revealed the influence of the small amount of polysaccharide additive since SA enhanced the microstructure, favored CB embedding, improved continuity of the 3D printed filaments, increased elasticity during compression, improved the overall mechanical properties, ensured beneficial effects in terms of cell–scaffold interactions, favored mineralization during MC3T3-E1 murine preosteoblast-containing CM incubation. These results indicate the capacity of marine-origin biomaterials to be used as precursors to develop 3D-printed scaffolds with bone regenerative potential.

## Figures and Tables

**Table 1:** Final mass ratio of the components in the formulated inks.

Code	Mass Ratio in the Final Mixture		
	FG	SA	CB
GCB	27.78	-	72.22
GA0.5CB	27.74	0.14	72.12
GA1CB	27.7	0.28	72.02
GA1.5CB	27.66	0.42	71.92

**Table 2:** Established processing parameters after printing experiments.

Samples Code	Pressure (kPa)	Feed Rate (mm/s)
GCB	550 ± 50	3.3–3.5
GA0.5CB		2.9–3.1
GA1CB		2.5–2.7
GA1.5CB		1.8–2.2

## Data Availability

Not applicable.

## Supplementary Materials

Videos S1–S4: Microstructural details visualized by micro-CT videos: S1 (GA0.5CB), S2 (GA1CB), S3 (GA1.5CB), and S4 (GCB). (available at https://zenodo.org/deposit/7086397 accessed on 16 September 2022). Videos S5–S8. Microstructural details of samples deformation under uniaxial compression at various compression points 0 mm (grey), 0.5mm (red), 1 mm (green) and 1.5 mm (blue): S5 (GA0.5CB), S6 (GA1CB), S7 (GA1.5CB), and S8 (GCB). (available at https://zenodo.org/deposit/7086397 accessed on 16 September 2022).
